# Investigating surface proteins and antibody combinations for detecting circulating tumor cells of various sarcomas

**DOI:** 10.1038/s41598-024-61651-w

**Published:** 2024-05-29

**Authors:** Minh-Chau N. Le, Kierstin A. Smith, Pablo J. Dopico, Beate Greer, Morteza Alipanah, Yang Zhang, Dietmar W. Siemann, Joanne P. Lagmay, Z. Hugh Fan

**Affiliations:** 1 Interdisciplinary Microsystems Group, Department of Mechanical and Aerospace Engineering, University of Florida, PO Box 116250, Gainesville, FL 32611 USA; 2 Department of Pediatrics, Division of Hematology-Oncology, University of Florida, Gainesville, FL 32610 USA; 3 Department of Radiation Oncology, University of Florida, Gainesville, FL 32610 USA; 4 J. Crayton Pruitt Family Department of Biomedical Engineering, University of Florida, Gainesville, FL 32611 USA

**Keywords:** Cancer, Health care, Oncology, Chemistry, Engineering

## Abstract

Circulating tumor cells (CTCs) have gathered attention as a biomarker for carcinomas. However, CTCs in sarcomas have received little attention. In this work, we investigated cell surface proteins and antibody combinations for immunofluorescence detection of sarcoma CTCs. A microfluidic device that combines filtration and immunoaffinity using gangliosides 2 and cell surface vimentin (CSV) antibodies was employed to capture CTCs. For CTC detection, antibodies against cytokeratins 7 and 8 (*CK*), pan-cytokeratin (*panCK*), or a combination of *panCK* and *CSV* were used. Thirty-nine blood samples were collected from 21 patients of various sarcoma subtypes. In the *independent samples* study, samples were subjected to one of three antibody combination choices. Significant difference in CTC enumeration was found between *CK* and *panCK* + *CSV*, and between *panCK* and *panCK* + *CSV*. Upon stratification of *CK*^+^ samples, those of metastatic disease had a higher CTC number than those of localized disease. In the *paired samples* study involving cytokeratin-positive sarcoma subtypes, using *panCK* antibody detected more CTCs than *CK*. Similarly, for osteosarcoma, using *panCK* + *CSV* combination resulted in a higher CTC count than *panCK*. This study emphasized deliberate selection of cell surface proteins for sarcoma CTC detection and subtype stratification for studying cancers as heterogeneous as sarcomas.

## Introduction

Sarcomas are a heterogeneous group of relatively rare, but lethal, tumors of mesenchymal origin^1,2^. Although they make up only 1% of all adult malignancies, sarcomas account for about 7% of childhood tumors^3^. Moreover, the 5 year survival rate for pediatric sarcomas ranges from 73% to a mere 45%, depending on the subtype of sarcomas^3^. The term ‘sarcoma’ represents over fifty subtypes of bone (e.g., osteosarcoma and Ewing) and soft tissue (e.g., rhabdomyosarcoma) malignant tumors that can be further categorized by their molecular pathology^1,4^. While the onset of carcinomas can often be pinpointed to the formation of precursor lesions, the origin of sarcomas and their various molecular features remain poorly understood^5^. Therefore, despite advancements in diagnostic imaging modalities, surgical techniques, and systemic chemotherapies, sarcoma patients with recurrent or metastatic disease still face a grim outcome^2^.

Circulating tumor cells (CTCs) are tumor cells that have shed from the primary tumor and entered the circulatory system to initiate the metastatic process^6^. With the appearance of 1–10 CTCs per 1 mL of blood, they are a rare, but an accessible biomarker for studying cancer metastasis^7–9^. For carcinoma CTC detection, multiplexed immunofluorescence staining remains a popular approach^10,11^. The FDA-approved CTC isolation system, CellSearch, utilizes immunofluorescent staining for cytokeratins (CK) 8, 18, and 19, the absence of the common leukocyte antigen CD45, and the nuclear dye DAPI, after isolation using antibody against epithelial cell adhesion molecule (EpCAM)^12^. Extensive research efforts showing the clinical implications of CTCs in carcinomas (e.g., breast, pancreatic, lung) have long been initiated^13–18^.

Unfortunately, the attention given to CTCs in sarcomas is severely lacking compared to other cancers^19^. Many studies reported in literature detected sarcoma CTCs using polymerase chain reaction (PCR)-based methods^19,20^. However, PCR-based approaches have several limitations, including the dependence of sensitivity on the expression level of the targeted transcript, the lack of CTC visualization, and unreliable CTC enumeration^21^. CTC detection methods based on immunocytochemistry/immunofluorescence labeling, though not without their limitations^10^, offers complementary advantages such as CTC morphology visualization, enumeration, and immunomolecular analysis^21–25^. One notable work on sarcoma CTCs detection using immunofluorescence was reported recently, in which the commercially available Abnova Cytoquest was used to isolate cell-surface vimentin (CSV)-positive CTCs (defined as CSV^+^CD45^−^) from pediatric sarcoma patients to assess overall survival^26^.

Although the heterogeneity of sarcomas has been well-documented^27^, its rarity limits the establishment of in-depth studies on individual subtypes. Many of the studies on detection of sarcoma CTCs via immunocytochemistry/immunofluorescence have focused on either proving the efficacy of one universal marker^26,28^ or comparing between an epithelial and a mesenchymal marker in a cohort of widely diverse sarcoma subtypes^21,29^. To the best of our knowledge, no work has been reported on comparing surface protein selections for CTC detection in specific sarcoma subtypes. Previously, our research lab has studied a lateral filter array microfluidic (LFAM) device functionalized with antibodies against gangliosides 2 (GD2) and CSV for isolating various cultured osteosarcoma (OS) cells spiked into a buffer or healthy blood samples and for detecting CTCs in two OS patient samples^30^. In this work, we studied the effect of surface protein selection on sarcoma CTC enumeration in peripheral blood samples of a variety of sarcoma patients. To account for the heterogeneity of sarcomas, we employed antibodies targeting three choices of cell surface proteins: cytokeratin 7/8 (*CK*), pan-cytokeratin (*panCK*), and a combination of *panCK* and *CSV* (*panCK* + *CSV*). With a total cohort of 21 patients from seven sarcoma subtypes, we conducted several studies utilizing different subsets of patients. First, we selected a subset of patients for an *independent samples* study, and randomly subjected their blood samples to one of the three marker selections to reveal trends in CTC enumeration. Then, motivated by the subtype diversity and availability of patients, we focused on three sarcoma subtypes–synovial sarcoma (SS), desmoplastic small round cell tumor (DSRCT), and OS. On these subtypes and their respective subsets of patients, we performed *paired samples* studies and found statistically significant difference between the cell surface proteins for CTC detection. Our work highlighted the importance of sarcoma subtype-based stratification and surface protein selection for studying sarcoma CTCs.

## Results

### Effect of cell surface proteins on CTC enumeration

Eighteen sarcoma patients provided a total of 27 samples that were subjected to processing as *independent samples* (see “Sample processing workflow” of “Methods” section). In this subset of 18 patients, some patients were sampled at more than one timepoint (see “Patient sample collection” of “Methods” section), resulting in a total of 27 samples that included 11 OS, seven Ewing sarcoma (EWS), four rhabdomyosarcoma (RMS), one chordoma, one DSRCT, one round cell sarcoma (RCS), and two SS (Supplementary Table S1). From three samples of healthy subjects, a baseline value of 0 CTC per mL for all three marker combinations was established. In this study, 17 out of 27 (63%) patient samples were positive for CTCs.

**Figure 1:**
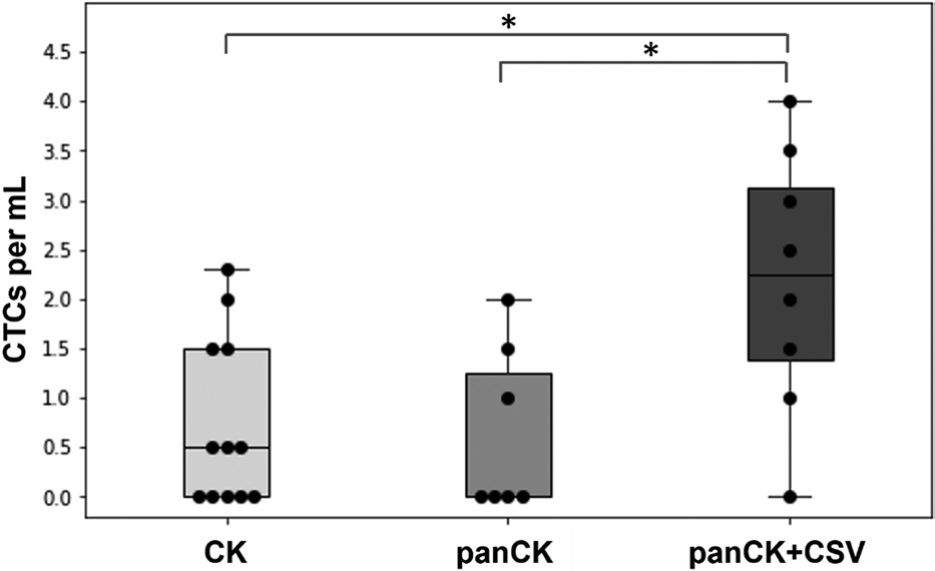
CTC enumeration of patient samples subjected to only one of three marker combinations: *CK* (n = 12), *panCK* (n = 7), or *panCK* + *CSV* (n = 8). For each box plot, sarcoma subtypes were pooled together. Statistical significance in CTC numbers were observed among the three groups using the Kruskal–Wallis test (p-value: 0.028). As indicated by the asterisks (*), post-hoc Dunn’s tests with Benjamini–Hochberg adjustments revealed significant differences between *CK* and *panCK* + *CSV* (p-value: 0.030), and between *panCK* and *panCK* + *CSV* (p-value: 0.030).

Tumor cells were captured in the microfluidic device using a combination of antibodies against GD2 and CSV. The isolated cells in the devices were randomly subjected to one of three immunofluorescence staining combinations for CTC detection (*CK*, *panCK*, or *panCK* + *CSV*) in addition to DAPI and CD45 staining. Figure 1 presents the CTC enumeration data as categorized by the marker combination. Note that we report the number of CTCs per mL (CTCs/mL) of blood processed to reflect the variation in the volume of clinical samples we received. Hence, this is different from the absolute count of CTCs in 7.5 mL of blood samples used in the FDA-approved CellSearch platform and reported in many papers in the literature. The median CTCs/mL value detected by *CK*, *panCK*, and *panCK* + *CSV* were 0.5 (interquartile range (IQR) 1.5), 0.0 (IQR 1.25), and 2.25 (IQR 1.75), respectively. Using the Kruskal–Wallis test, a statistical difference (p-value: 0.028) was detected among the groups. Using the post-hoc Dunn’s test with Benjamini–Hochberg adjustments, statistical difference was detected between *CK* and *panCK* + *CSV* (p-value: 0.030), and between pan*CK* and *panCK* + *CSV* (p-value: 0.030). No significant difference was detected between *CK* and *panCK* in the post-hoc multiple pairwise comparisons.

### Effect of disease status on CTC enumeration

**Figure 2:**
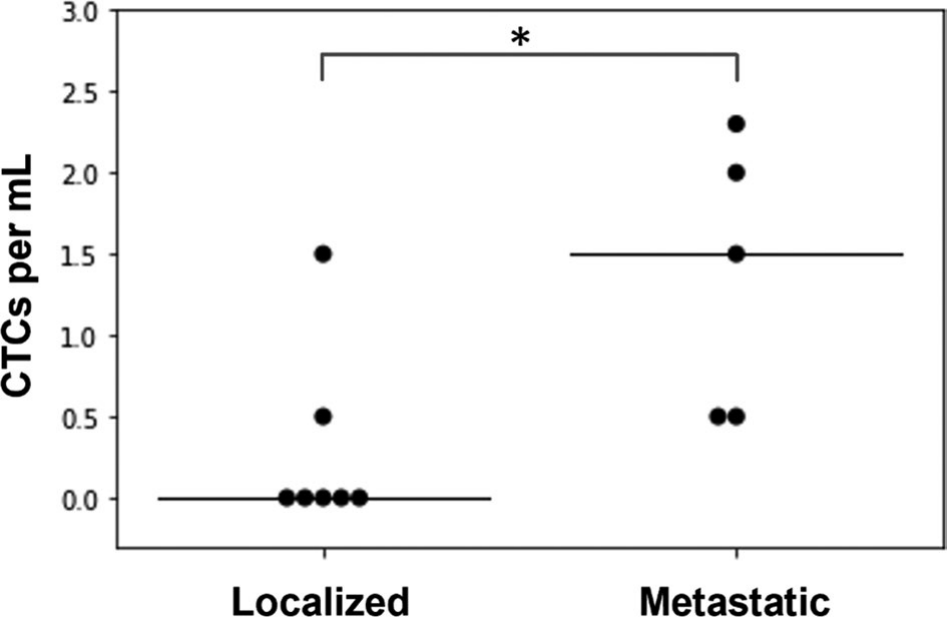
CTC enumeration data of the 12 sarcoma patient samples detected with *CK* as categorized by disease status. Each dot represents one patient sample. The black line indicates the median, while the asterisk (*) indicates statistically significant difference (p-value: 0.022) as determined by the Mann–Whitney U test. The statistically significant difference in CTC numbers between the disease status supports the hypothesis of tumors shedding CTCs into the blood during cancer metastasis.

To assess the effect of disease status (localized or metastatic) on CTC enumeration, the *independent samples* that were stained with *CK* were stratified based on their disease status regardless of the sarcoma subtype. Ten sarcoma patients provided 12 samples for this analysis (Supplementary Table S2). Of the 12 samples, seven came from patients with localized disease (i.e., localized samples) and five came from patients with metastatic disease (i.e., metastatic samples). The median CTCs/mL value for *localized* samples was 0.0 (IQR 0.25), while that for *metastatic* samples was 1.5 (IQR 1.5) (Fig. 2). Using the Mann–Whitney U test, a statistically significant difference (p-value: 0.022) was detected between the median CTC numbers of the localized and metastatic samples. Taking a closer look at the CTC detection rates, CTCs were detected in two out of seven (29%) localized samples, but in all five (100%) metastatic samples.

### Effect of CK antibody clones on CTC enumeration in SS and DSRCT

**Figure 3:**
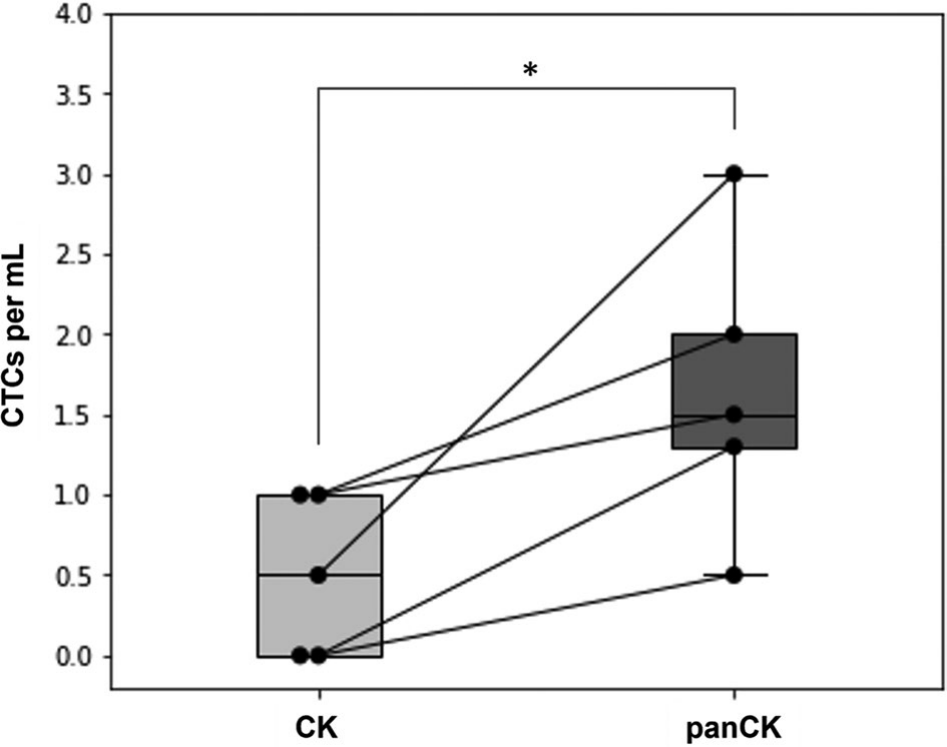
CTC enumeration for CK-positive sarcomas (SS and DSRCT) using *CK* and *panCK* surface marker combinations. The paired boxplot shows the spread of the CTC numbers categorized by the staining method. Each dot represents the CTC number of one patient sample. Paired data points (i.e., *paired samples*) are connected by a line. The (*) indicates statistically significant difference in CTC number between the two marker combinations, as determined by the Wilcoxon Signed-Rank test (p-value: 0.042).

For this study, five blood samples were collected from three SS patients and one DSRCT patient (Supplementary Table S3), without regard to the disease status (i.e., localized, or metastatic) of the patients. Blood samples were subjected to *paired samples* processing (see “Sample processing workflow” of “Methods” section), in which one blood sample was evenly split into two portions for processing by two microfluidic devices, with one microfluidic device subjected to *CK*, and the other to *panCK*. CTCs were detected in three out of five (60%) samples using *CK*, but in all (100%) samples using *panCK* (Fig. 3). The median CTCs/mL using *CK* and *panCK* were 0.5 (IQR 1) and 1.5 (IQR 0.7), respectively. Using the Wilcoxon Signed-Rank test, statistically significant difference in median CTC number between *CK* and *panCK* was detected (p-value: 0.042).

### Effect of CSV detection on CTC enumeration in OS

**Figure 4:**
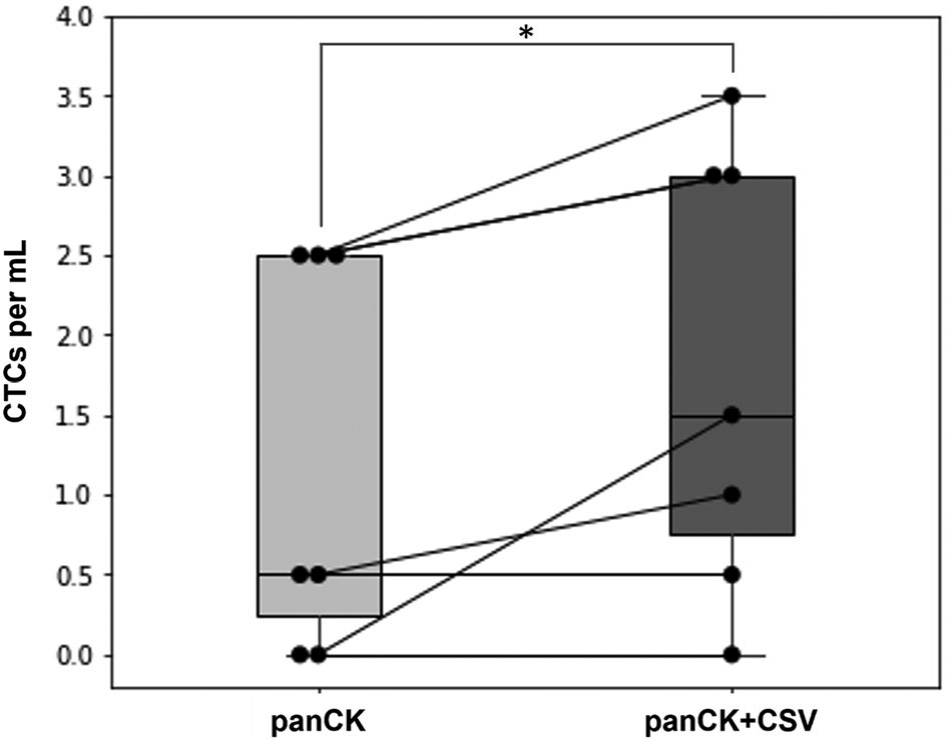
CTC enumeration for OS samples using *panCK* and *panCK* + *CSV* marker combinations. Paired data points are connected by a line. The (*) indicates statistically significant difference (p-value: 0.039) in CTC number between the two marker combinations, as determined by the Wilcoxon Signed-Rank test.

The second *paired samples* study investigated the effect of adding antibodies against CSV for OS CTCs detection. Seven blood samples, regardless of disease status, were collected from six OS patients (Supplementary Table S4). Blood samples were subjected to *paired sample* processing to compare the two marker combinations: *panCK* and *panCK* + *CSV*. CTCs were detected in five out of seven (71%) samples using *panCK*, and in six out of seven (86%) samples using *panCK* + *CSV* (Fig. 4). A statistically significant difference in median CTC number between the marker combinations was detected (p-value: 0.039) using the Wilcoxon Signed-Rank test. The median CTC/mL values for *panCK* and *panCK* + *CSV* were 0.5 (IQR 2.25) and 1.5 (IQR 2.25), respectively.

## Discussion

As the attention on sarcoma CTCs is still lacking compared to carcinoma CTCs, it becomes necessary to explore surface proteins appropriate for the immunofluorescence detection of sarcoma CTCs. In our study, a microfluidic platform was used to detect CTCs from the blood samples of sarcoma patients. The platform device integrated immunoaffinity-based isolation with filtration-based capture to address CTC’s heterogeneity in both size and the expression level of surface protein markers^31^. By current knowledge, one of the ways by which cancer metastasizes is through the intravasation of CTCs into the circulatory system, its survival in the bloodstream, extravasation, and potential colonization of the distant site^32^. While it is estimated that thousands of CTCs are released into the bloodstream each day^33^, a very low concentration of CTCs is often detected^34^, denoting the inefficiency of this metastasis process. For our patient cohort, we reported a range of 0 to 4 CTCs/mL, a range in line with those reported in literature for sarcoma CTCs detection^26,29,35^.

The understanding of CTCs being biomarkers of disease progression was reflected in our study. When we stratified the *independent samples* that were stained with *CK* by their disease status, higher CTC detection rate and higher CTC numbers were found in *metastatic* samples compared to localized samples (Fig. 2). In a study with lung cancer patients, a similar trend was observed where increased disease staging showed increased average CTC numbers^36^. A high CTC detection rate in metastatic disease has also been reported elsewehere for carcinomas such as ovarian cancer^37^, non-small cell lung cancer^38^, and esophageal cancer^39^. The CTC detection rate for localized diseased samples, although lower than that of metastatic diseased samples, is comparable to those reported for non-metastatic esophageal cancer (15.8%)^39^ and primary breast cancer (20.2%)^40^. The detection of CTCs in non-metastatic disease reported in these studies on carcinomas^39,40^, as well as the results reported in our study for localized sarcomas, reflect the parallel cancer progression model. According to parallel progression model, tumor cells are disseminated early on in the disease progression sometimes even before the primary tumor is clinically detectable^41,42^.

The *independent samples* study involved three combinations of surface proteins for CTC detection and seven sarcoma subtypes. Not only did Fig. 1 agree with intuition, as a more inclusive tumor cell marker combination (i.e., *panCK* + *CSV*) would detect more CTCs, but it also aligned with the general research findings reported in literature regarding the expressions of CKs and CSV in sarcomas. *CK* (clone: CAM 5.2) recognizes CK 7 and CK 8 antigens present on simple epithelia^43^. *PanCK* (clone: AE1/AE3) recognizes a wider selection of both acidic and basic CKs to cover both simple and stratified epithelia, and epidermis^43^. More specifically, AE3 monoclonal antibody (mAb) identifies CK1 through 8, and AE1 mAb recognizes CK10, 13, 14, 15, 16, and 19^44^. CSV (clone: 84-1) has been reported as a universal surface marker on sarcoma CTC, regardless of the sarcoma tumor origin^28^. Piecing these research findings in literature together, one could comprehend how *panCK* + *CSV* essentially covers CTCs from all sarcoma subtypes to give a higher CTC count compared to only having *CK* or *panCK* staining.

However, Fig. 1 also depicted a rather interesting phenomenon regarding both the CTC counts and the detection rates for each cell marker combination. The detection rates for *CK*, *panCK*, and *panCK* + *CSV* were seven out of 12 (58%), three out of seven (43%), and seven out of eight (88%), respectively. Based on the reported range of markers that each staining method would recognize, *panCK* would potentially recognize CTCs more readily than *CK*. However, this was not observed in the data as there was no statistically significant difference observed in the CTC counts between *panCK* and *CK.* Furthermore, the detection rate for *CK* was higher than that of *panCK*. This unexpected observation in the *independent samples* study sparked the question of whether pooling together different sarcoma subtypes would pose a clinically meaningful comparison for a sarcoma marker study.

The notable challenges in advancing diagnostics and therapeutics for sarcomas is rooted in not only its rarity^45^, but also in the complexity of each subtype^19^. According to the World Health Organization (WHO), sarcomas are further subcategorized into more than 70 distinctive subtypes^46^. The heterogeneity of sarcomas, and consequently, the wide range of detected CTCs were also reflected elsewhere in literature. In a pioneering sarcoma CTC work conducted by Paterlini-Bréchot and colleagues, multi-color immunocytochemistry were used for CTC detection following the filtration-based isolation of CTCs^21^. CTCs were identified as either vimentin^+^CD45^−^CD34^−^ or panCK^+^CD45^−^CD34^−^. In a small cohort consisting of 11 patients representing six subtypes of sarcomas, a wide range of 2.0 to 48.0 CTCs per eight mL of blood, which was equivalent to a range of 0.25 to 6.0 CTCs/mL, was reported. However, no distinct pattern in CTC enumeration was detected within or across the different subtypes. Similarly, in a later work consisting of 36 sarcoma patients from four subtypes, Loeb and collaborators reported no differentiating characteristic between newly diagnosed patients with low CTC counts and those with high CTC counts despite a clear bimodal distribution of CTC enumeration^47^. In this study, the employment of the CellSieve™ microfiltration system and a vimentin^+^CD45^−^ CTC definition resulted in a wide range of 0.0 to 51.4 CTCs/mL and an overall detection rate of 65% – a rate similar to that of our *independent samples* study. Compared to our work, the upper limits of CTC number reported in these two publications are higher. However, a closer look at the patients with the highest CTC numbers in these two publications revealed that most were stage-IV^21^ or newly diagnosed (i.e., prior to any treatment)^47^, characteristics that could result in elevated CTC counts. In contrast, patients in our study were recruited without regard to staging or treatment status. From our own observations in Fig. 1 and the literature, we can infer that to extract any meaningful clinical utility of sarcoma CTCs, it is necessary to conduct subtype-specific CTC detection and analysis using *paired samples*.

The first *paired samples* study focused on the effect of CKs on the detection of CTCs in SS and DSRCT. CK expression has been documented in sarcomas such as SS (CK7 and CK19)^48,49^, DSRCTs (a variety of CKs except for CK20, 5, and 6)^48^, rare cases of OS^50^, and aberrantly in EWS and RMS (CK8 and 18)^48,51^. Since the expression of CKs in sarcomas is reportedly consistent in SS and DSRCT, but anomalous in other subtypes, SS and DSRCT were chosen as the target subtypes of this *paired samples* study.

Initially, Fig. 1 showed no statistically significant difference in CTC count between *CK* and *panCK* when seven sarcoma subtypes with different *CK* expressions were studied. However, when we considered only two sarcoma subtypes positive for *CK* (Fig. 3), different observations were obtained. In this *paired samples* study, *panCK* resulted in both a higher CTC detection rate and a higher CTC enumeration than *CK*. These results clearly support the importance of subtype-specific approach for studying cancers as heterogeneous as sarcomas.

This first *paired samples* study also contributes to the general growing understanding of SS and DSRCT CTCs. Studies on CTCs in SS and DSRCT as prognostic tools have remained scarce due partly to the limited number of patients available for study^19^. SS is the more studied subtype of the two, possibly due to its tendency towards hematogenous spread^52^. Sapi and colleagues stated the rarity of CTCs in SS after attempting to detect the SS18-SSX fusion genes in the blood of 15 patients^49^. Paterlini-Bréchot and her research group detected CTCs in all of the six SS patients being studied using the ISET (isolation by size of tumor cells) system and defining CTCs as either vimentin^+^CD45^−^CD34^−^ or panCK^+^CD45^-^CD34^−^^21^. Here, we employed a microfluidic device featuring both filtration and immunoaffinity-based capture mechanisms to successfully isolate CTCs in both SS and DSRCT.

The second *paired samples* study focused on the subtype OS. In literature, several works had demonstrated the diagnostic and prognostic value of CTCs in OS. While Wong et al. used reverse-transcription PCR to correlate elevated levels of CTC-originated *COLL* mRNA in peripheral blood to metastasis^53^, Zhang et al. used fluorescence in situ hybridization to establish a cut-off CTC value for progression-free survival^54^. This *paired samples* study contributes to the use of immunofluorescence in OS CTCs detection by showing how adding *CSV* results in a higher CTC enumeration compared to just using *panCK* (Fig. 4). Previously, we had shown a low expression of EpCAM across all the OS cell lines tested, and a high expression of CSV across those same OS cell lines^30^. In a study that compared CTC detection rates of EpCAM and CSV as capture antibodies for sarcoma patient samples, Kang and collaborators reported a 0% rate for EpCAM, but a 90% rate for CSV^29^. These observations were also reflected elsewhere in the literature where strong immunoreaction for the epithelial CKs was reported rare for OS^50^. Another study also showed the use of CSV in labeling tumor cells from OS cell lines and OS patient-derived xenograft for imaging mass cytometry-based protein profiling^55^. Here, we have demonstrated the use of anti-CSV antibodies for immunofluorescence-based CTC detection specifically in OS patient samples, thus, contributing to the growing understanding of CSV expression in OS CTCs.

In summary, we have compared the CTC enumeration and detection rates of three combinations of surface protein markers across various sarcoma subtypes. Furthermore, our work has emphasized the importance of subtype stratification in studying sarcoma CTCs. The immediate next step would be the downstream analysis (e.g., proteomic profiling, RNA sequencing) of the detected sarcoma CTCs, which could be investigated by releasing the captured CTCs from the microfluidic device as previously demonstrated^31^. However, in this current study, there were several limitations. Firstly, there were challenges with the availability of new sarcoma patients, especially for some rarer subtypes such as synovial sarcomas and desmoplastic small round cell tumor. Therefore, some patients were sampled more than once in a study. In the future, patient recruitment could either be expanded to multiple medical centers to ensure a more robust cohort or be targeted towards subtypes that are abundantly available on-site. Secondly, due to our current fluorescence imaging system having only three fluorescence filters (DAPI, RFP, and GFP), our number and choice of fluorophores for this study were limited. Therefore, for the *paired samples* study comparing *panCK* and *panCK* + *CSV* for OS CTC detection, the fluorophores in Alexa Fluor 488-labeled antibodies against panCK (panCK-AF) and fluorescein-isothiocyanate-labeled antibodies against CSV (CSV-FITC) have nearly identical emission maxima (Supplementary Fig. S1). Thus, differentiating between *panCK* and *CSV* and characterizing the expression of either protein on a detected CTC were difficult. Future experiments could consider expanding the range of available imaging light source and filters to ensure a well-selected combination of fluorophores for multi-color immunofluorescence CTC detection.

## Methods

### Reagents and buffers

Biotinylated antibodies against GD2 (clone: 14G2a) were obtained from BioLegend (San Diego, CA) while biotinylated antibodies against CSV (clone: 84-1) were from Abnova (Taipei, Taiwan); they were immobilized on the surface of the microfluidic channels to target the tumor cells. CK-FITC (clone: CAM 5.2, BD Biosciences, Franklin Lakes, NJ), panCK-AF (clone: AE1/AE3, eBiosciences, San Diego, CA), and CSV-FITC (clone: 84-1, Abnova, Taipei, Taiwan) were used in the immunofluorescence staining of the tumor cells. Phycoerythrin-labeled antibodies against CD45 (CD45-PE, BD Biosciences, Franklin Lakes, NJ) were used for the immunofluorescence staining of leukocytes. DAPI (4′,6-Diamidino-2-Phenylindole, Dilactate, Invitrogen, Waltham, MA) was used to counterstain the nuclei of nucleated cells captured in the device. Dulbecco’s phosphate-buffered saline without calcium and magnesium (DPBS, Cytiva, Marlborough, MA) was used for washing during cell preparation, device functionalization, sample processing, and immunofluorescence staining. DPBS containing 2% bovine serum albumin (BSA, Fisher Scientific, Waltham, MA) was used as a blocking buffer during device functionalization and immunofluorescence staining.

### Microfluidic devices

Our research group has demonstrated the ability of the LFAM device to efficiently capture CTCs based on both filtration and immunoaffinity (Supplementary Fig. S2; Supplementary Table S5)^31^. Device fabrication and operation were as described previously^31^. To functionalize the microchannel surfaces with antibodies against GD2 and CSV, 100 µL of 2 mg/mL avidin (Invitrogen, Waltham, MA) in DPBS was loaded into the device, incubated for 15 min at room temperature to allow for the physical adsorption of avidin onto the microchannel surfaces, and washed with DPBS (1 µL/s, 250 µL). Then, 100 µL of biotinylated antibody combination containing 20 µg/mL biotinylated CSV and 20 µg/mL biotinylated GD2 in DPBS (*GD2* + *CSV*) was introduced into the device and allowed to incubate for 15 min at room temperature. Finally, the channels were rinsed with 2% BSA (1 µL/s, 250 µL) to remove excess antibodies. The 2% BSA was left incubated in the device for 20 min at room temperature before a sample was introduced into the device. This BSA solution was used as a blocking buffer to reduce non-specific absorption of other blood cells. In instances where the patient sample was not ready for processing immediately following device functionalization, the functionalized microfluidic devices were stored in a foil-wrapped humidified petri dish at 4 °C.

### Cell line

Human osteosarcoma Hu09 cells (CVCL-01298) were obtained from Dr. Lin Ren at the national cancer institute (NCI) as previously reported^30^. Cells were STR analyzed and authenticated. Cells were cultured in RPMI 1640 medium (Caisson Labs, Smithfield, UT) supplemented with 10% fetal bovine serum (Corning, Smithfield, UT) and 1% penicillin-streptomycin (Sigma-Aldrich, St. Louis, MO), and incubated at 37 °C with 5% CO_2_ in a humidified incubator. Cells received frequent media changes and were expanded to 80% confluency before being detached with 0.25% trypsin-ethylenediaminetetraacetic acid (0.25% trypsin-EDTA, 1X, phenol red, Caisson Labs, Smithfield, UT) for experiments or sub-culturing at a lower concentration. Mycoplasma tests were carried out to monitor potential cell culture contaminations. The cultured Hu09 cells were then subjected to immunofluorescence staining (see section “Immunofluorescence staining”) for establishing the imaging thresholds for CTC detection (see section “Fluorescence imaging and CTC enumeration”).

### Patient sample collection

This study was approved by the university of florida institutional review board (IRB) under IRB#201900786 and the study methodologies conformed to the standards set by the Declaration of Helsinki. A total of 39 blood samples were received from 21 patients with different sarcoma subtypes and disease statuses after written informed consent was obtained from the participants and/or their legal guardians. The cohort consisted of seven OS patients, five EWS patients, two RMS patients, one chordoma patient, one DSRCT patient, one RCS patient, and four SS patients. The cohort included patients who were actively undergoing standard treatment plan according to their subtype and condition, and those who were visiting the clinic for off-treatment follow-up. Characteristics of the 21 patients participating in this work are summarized in Table 1. Some patients contributed multiple samples to the study (Supplementary Table S6), with the time difference between adjacent samples ranging between 3 weeks and 15 months. For each patient, the timepoint of the blood draw was not determined by their treatment status. Rather, the timepoint was determined by the patient’s appointment schedule and whether they were well enough to give blood as determined by the physician. Therefore, this study included blood samples that were randomly drawn both during active treatment and off-treatment follow-up.

**Table 1:** Patient characteristics (n = 21).

Characteristic	Number of patients (%)
Age	
Age $\le$ 10	3 (14%)
Age $>$ 10	18 (86%)
Biological sex	
Male	17 (81%)
Female	4 (19%)
Race	
White	19 (90%)
Black or African American	2 (10%)
Ethnicity	
Non-hispanic	19 (90%)
Hispanic or latino	2 (10%)
Sarcoma subtype	
Osteosarcoma	7 (33%)
Ewing sarcoma	5 (24%)
Synovial sarcoma	4 (19%)
Rhabdomyosarcoma	2 (9%)
Chordoma	1 (5%)
Round cell sarcoma	1 (5%)
Desmoplastic small round cell tumor	1 (5%)

Blood (3–6 mL per sample) was drawn into Vacutainer tubes (BD, Franklin Lakes, NJ) containing K_2_-EDTA. Blood samples were de-identified of patient information before sending to the researchers. Samples were stored at 4 °C until sample processing. To establish a baseline value for CTC count, human whole blood samples from healthy donors were purchased from Innovative Research, Inc. (Novi, MI).

### Patient sample processing

All sarcoma patient samples were processed within 48 h of sample collection. Purchased healthy blood samples were processed on the day of receipt. Blood was diluted with DPBS at a 1:1 ratio to create a 2X dilution of whole blood as previously described^30^. Diluted blood, along with a 5-by-2 mm magnetic stir bar (Fisher Scientific, Waltham, MA), were loaded into a 5 mL BD Luer Lok syringe (BD, Franklin Lakes, NJ). The syringe was equipped with a barbed luer adapter (IDEX, Northbrook, IL) and a chromatography tubing piece (IDEX, Northbrook, IL) that was connected to the inlet of the microfluidic device. The syringe, luer adapter, and tubing were pretreated with 2% BSA prior to handling the blood sample to reduce non-specific absorption. About 4 mL of diluted blood (i.e., 2 mL of whole blood mixed with 2 mL of DPBS) was passed through a functionalized LFAM device using a syringe pump (1 µL/s). During this sample passaging step, a magnetic stir plate was placed underneath the syringe to enable the magnetic stir bar to keep the blood suspended. After the passaging of blood, DPBS (2 µL/s, 450 µL) was introduced into the device to wash out non-specifically adhered cells. Devices were stored in a foil-wrapped humidified petri dish at 4 °C until ready for immunofluorescence staining and imaging.

### Immunofluorescence staining

After the passaging of blood in the LFAM device, cell fixation using 4% paraformaldehyde (1 µL/s, 150 µL) and permeabilization using 0.2% Triton X-100 (1 µL/s, 150 µL) were done successively, each with a 10 mins incubation time followed by a DPBS wash (2 µL/s, 250 µL). Then, 2% BSA (1 µL/s, 150 µL) was added to the device as the blocking buffer, and incubated for 30 mins. To identify the targeted CTCs among the non-targeted white blood cells (WBCs) captured in the device, a cocktail consisting of fluorophore-conjugated antibodies was introduced into the device. The 100 µL cocktail consisted of 2% BSA, CD45-PE (20 µL, volume per test recommended by manufacturer), and one of the following three tumor marker choices: (1) 2.5 µg/mL CK-FITC, (2) 2 µg/mL panCK-AF, or (3) a combination of 2 µg/mL panCK-AF and 2 µg/mL CSV-FITC. The cocktail was incubated for 1 h, followed by a DPBS rinse (2 µL/s, 450 µL). Finally, the cells were counterstained with 300 µM DAPI for 10 mins and washed with DPBS (2 µL/s, 450 µL). All immunofluorescence staining steps were conducted at room temperature. The three marker combinations were referred to as (1) *CK*, (2) *panCK*, and (3) *panCK* + *CSV*, respectively. Based on these three marker combinations, the following three phenotypes were identified as CTCs in this study: DAPI^+^CK^+^CD45^−^, DAPI^+^panCK^+^CD45^−^ and DAPI^+^(panCK + CSV)^+^CD45^−^.

### Fluorescence imaging and CTC enumeration

A Lionheart LX automated microscope (Agilent, Santa Clara, CA), equipped with three sets of LED and filter cubes for multi-channel image capture, was used to take fluorescence images of the entire microfluidic device. Its accompanying software, the Gen5 microplate reader and Imager software (Agilent, Santa Clara, CA), was used to program automatic device imaging and subsequent image analysis. A device layout mapping the boundaries of the microchannels was designed in Gen5. Based on the fluorophores used in immunofluorescence staining, automated imaging using a 4X objective was conducted in four color channels: brightfield, DAPI, RFP (for CD45-PE), and GFP (for CK-FITC, panCK-AF, and CSV-FITC). After image capture, background noise removal was performed using the rolling-ball algorithm provided in Gen5.

**Figure 5:**
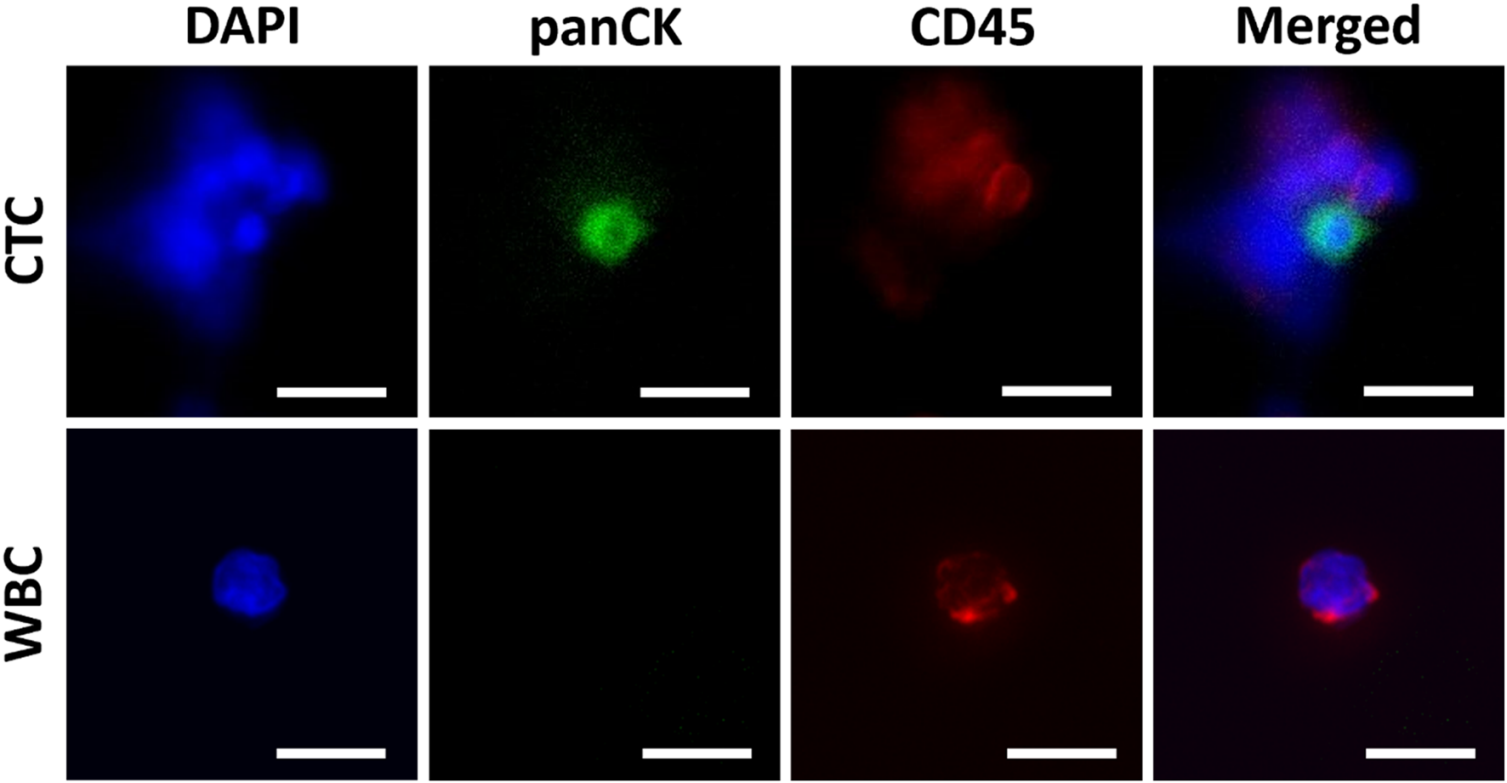
A CTC and a WBC as detected using immunofluorescence staining and imaging. In this example, the patient sample (patient ID: 008) was subjected to the fluorescence cocktail consisting of DAPI, panCK-AF, and CD45-PE. The CTCs were defined by the phenotype DAPI^+^panCK^+^CD45^−^. The WBCs were defined as DAPI^+^panCK^−^CD45^+^. Scale bars are 20 µm.

For image analysis, a pipeline was set up in Gen5 to automatically identify DAPI^+^GFP^+^RFP^−^ regions as “potential CTCs”. Using the analysis pipeline, images in the GFP channel were first analyzed to identify signals meeting the thresholds for GFP intensity, size, and circularity. Thresholds for such parameters were determined from fluorescence images of cultured Hu09 cells subjected to the same microfluidic processing and immunofluorescence staining procedures described above. Then, the identified GFP-positive regions were further checked for presence of DAPI and absence of RFP according to the signal thresholds set using fluorescence images of cultured Hu09 cells as described above, and of leukocytes in clinical samples subjected to the established processing and immunofluorescence staining procedures, respectively. Images from regions meeting the “potential CTC” criteria of DAPI^+^GFP^+^RFP^−^ were overlaid to attain the merged images (Supplementary Fig. S1). Finally, the individual and merged images of these “potential CTCs” were manually inspected for relative positions and shapes of the fluorescence signals before being counted as a CTC. For “potential CTCs” needing a closer inspection, images at 20× (Fig. 5) were also taken before they were confidently confirmed as a CTC.

### Sample processing workflow

The volume of blood collected from each patient varied depending on the age and vasovagal response of the patient. If the sample came from a pediatric patient, the volume of blood obtained was often severely limited, with the most generous volume being enough for a maximum of two microfluidic devices. Depending on the sample volume collected, each sample could be processed by one or two microfluidic devices. In this study, samples processed by one device were referred to as *independent samples*, and those processed by two devices as paired samples.

**Figure 6:**
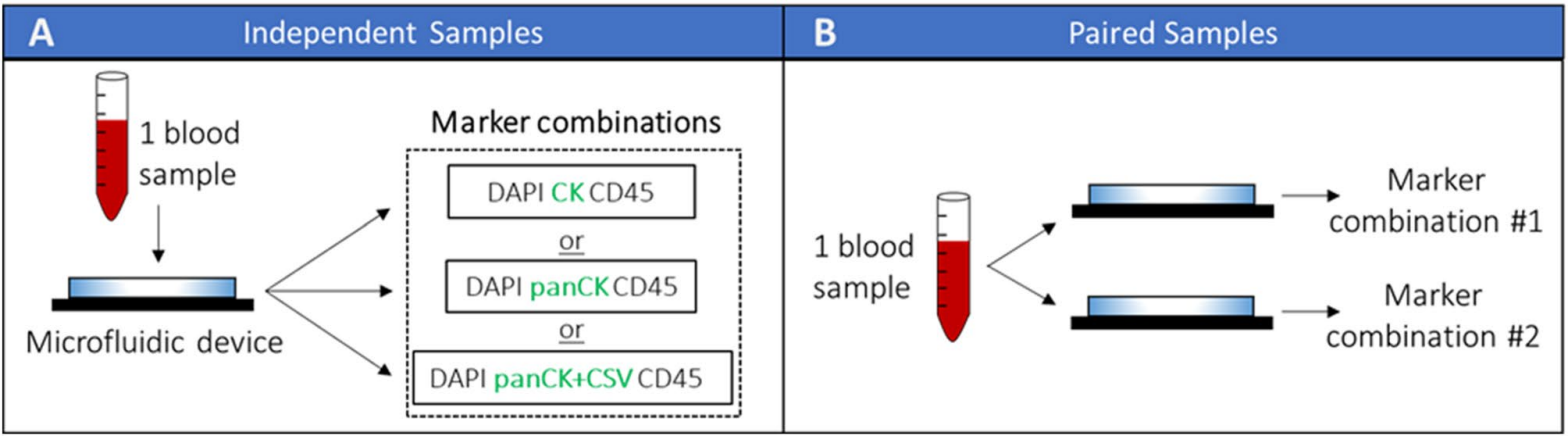
Schematic of sample processing workflow for *independent samples* and paired samples. (**A**) For experiments with *independent samples*, one blood sample was processed with one microfluidic device. This device was then subjected to a marker combination randomly selected from the three choices defined in this work. (**B**) For experiments with paired samples, one blood sample was evenly split into two portions for processing by two microfluidic devices, which were then subjected to two different marker combinations.

As shown in Fig. 6A, each *independent sample* was processed by one microfluidic device. This one microfluidic device was then subjected to one randomly selected marker combination out of the three options discussed above. Collectively, all *independent samples* in this project were analyzed for the effect of their respective marker combination selection on CTC enumeration. *Independent samples* subjected to the marker combination *CK* were used to study the effect of disease status (i.e., localized and metastatic) on CTC enumeration.

Each paired sample was processed by two microfluidic devices in parallel (Fig. 6B), with each device subjected to a different marker combination depending on the study of interest. To study how the inclusion of more CK subtypes affected CTC enumeration, paired samples were subjected to *CK* and *panCK* marker combinations. To study the effect of the mesenchymal marker CSV on CTC enumeration, *panCK* and *panCK* + *CSV* combinations were used.

### Statistical analysis

The small and different sample sizes in this study warranted careful considerations in the selection of applicable statistical tests^56^. Data was verified to have a non-normal distribution with equal variance using normal probability plots and Levene’s tests, respectively. Due to the non-normal data distribution, the center and spread of data were reported using the median and IQR values, respectively. For the same reason, non-parametric statistical tests were selected for this study. For detecting differences in the CTC counts of *independent samples*, the Kruskal–Wallis test followed by post-hoc Dunn’s test with Benjamini–Hochberg adjustments were used^57^. For analyzing the effect of disease status on CTC enumeration, the Mann–Whitney U test was applied. For paired samples studies, the Wilcoxon Signed Rank test was used. The Levene’s, Kruskal–Wallis, Mann–Whitney U, and Wilcoxon Signed Rank tests were conducted using the Python SciPy package (version 1.1.0). The post-hoc Dunn’s test was carried out using the Python scikit-posthocs package (version 0.7.0). A p-value < 0.05 was used in all statistical tests to demonstrate significant difference.

### Supplementary Information

Supplementary Information.

## Data Availability

The datasets generated during and/or analyzed during the current study are available from the corresponding author on reasonable request.
