# Supplementary material for: Investigating surface proteins and antibody combinations for detecting circulating tumor cells of various sarcomas
Source: Sci Rep. 2024 May 29;14:12374. doi: 10.1038/s41598-024-61651-w (PMC11137101; doi:10.1038/s41598-024-61651-w)
Supplement: Supplementary file 1 — Supplementary Information. [file 41598_2024_61651_MOESM1_ESM.pdf]

Supplementary Information

Investigating surface proteins and antibody combinations for detecting circulating tumor cells of various sarcomas

Minh-Chau N. Le[1], Kierstin A. Smith[1], Pablo J. Dopico[1], Beate Greer[2], Morteza Alipanah[1], Yang Zhang[1], Dietmar W. Siemann[3], Joanne P. Lagmay[2,*], and Z. Hugh Fan[1, 4, *]

[1]Interdisciplinary Microsystems Group, Department of Mechanical and Aerospace Engineering, University of Florida, Gainesville, FL 32611, USA

[2]Department of Pediatrics, Division of Hematology-Oncology, University of Florida, Gainesville, FL 32610, USA.

[3]Department of Radiation Oncology, University of Florida, Gainesville, FL 32610, USA

[4]J. Crayton Pruitt Family Department of Biomedical Engineering, University of Florida, Gainesville, FL 32611, USA

*Authors to whom correspondence should be addressed, email: hfan@ufl.edu, jplagmay@ufl.edu.

Table S1. Characteristics of the 27 samples used for *independent samples* study.

Patient ID	Sample No.†	Sarcoma Subtype	Marker Combination	CTCs/mL
001	#1	Osteosarcoma	*CK*	0.0
001	#2	Osteosarcoma	*panCK+CSV*	3.0
002	#1	Rhabdomyosarcoma	*CK*	2.3
002	#2	Rhabdomyosarcoma	*panCK*	0.0
003	#1	Chordoma	*CK*	0.5
004	#1	Rhabdomyosarcoma	*CK*	0.5
004	#2	Rhabdomyosarcoma	*CK*	0.0
005	#1	Osteosarcoma	*CK*	1.5
005	#2	Osteosarcoma	*CK*	0.0
007	#1	Osteosarcoma	*CK*	0.0
007	#2	Osteosarcoma	*panCK+CSV*	2.0
007	#4	Osteosarcoma	*panCK+CSV*	3.5
008	#1	Osteosarcoma	*panCK*	2.0
008	#2	Osteosarcoma	*panCK*	1.5
013	#1	Round Cell Sarcoma	*CK*	0.5
014	#1	Ewing Sarcoma	*CK*	0.0
015	#1	Ewing Sarcoma	*CK*	1.5
016	#1	Ewing Sarcoma	*CK*	2.0
016	#2	Ewing Sarcoma	*panCK+CSV*	0.0
017	#1	Ewing Sarcoma	*panCK*	0.0

Table S1 (cont'd).

**Patient ID**	**Sample No.**†	**Sarcoma Subtype**	**Marker Combination**	**CTCs/mL**
018	#1	Osteosarcoma	*panCK*	0.0
019	#1	Ewing Sarcoma	*panCK+CSV*	2.5
019	#2	Ewing Sarcoma	*panCK+CSV*	1.0
022	#1	Synovial Sarcoma	*panCK+CSV*	4.0
025	#1	Desmoplastic Small Round Cell Tumor	*panCK+CSV*	1.5
026	#1	Synovial Sarcoma	*panCK*	0.0
041	#1	Osteosarcoma	*panCK*	1.0

†Some patients contributed more than one sample to the entire study. "Sample No." serves as an identifier to distinguish among different samples contributed by the same patient.

Table S2. Disease status of the 12 *independent samples* that were detected using *CK*.

Patient ID	Sample No.†	Sarcoma Subtype	Disease Status	CTCs/mL
001	#1	Osteosarcoma	Local	0.0
002	#1	Rhabdomyosarcoma	Metastatic	2.3
003	#1	Chordoma	Metastatic	0.5
004	#1	Rhabdomyosarcoma	Local	0.5
004	#2	Rhabdomyosarcoma	Local	0.0
005	#1	Osteosarcoma	Local	1.5
005	#2	Osteosarcoma	Local	0.0
007	#1	Osteosarcoma	Local	0.0
013	#1	Round cell sarcoma	Metastatic	0.5
014	#1	Ewing sarcoma	Local	0.0
015	#1	Ewing sarcoma	Metastatic	1.5
016	#1	Ewing sarcoma	Metastatic	2.0

†Some patients contributed more than one sample to the entire study. "Sample No." serves as an identifier to distinguish among different samples contributed by the same patient.

Table S3. Characteristics of the five samples collected for the *paired samples* study with CK-positive sarcomas (SS and DSRCT) using *CK* and *panCK* surface marker combinations.

**Patient ID**	**Sample No.**[†]	**Sarcoma Subtype**	***CK*** **CTCs/mL**	***panCK*** **CTCs/mL**
022	#2	Synovial Sarcoma	0.0	0.5
025	#2	Desmoplastic Small Round Cell Tumor	1.0	2.0
025	#3	Desmoplastic Small Round Cell Tumor	0.5	3.0
028	#1	Synovial Sarcoma	1.0	1.5
031	#1	Synovial Sarcoma	0.0	1.3

[†]Some patients contributed more than one sample to the entire study. "Sample No." serves as an identifier to distinguish among different samples contributed by the same patient.

Table S4. Characteristics of the seven samples collected for the *paired samples* study with OS patients using *panCK* and *panCK+CSV* surface marker combinations.

**Patient ID**	**Sample No.**[†]	**Sarcoma Subtype**	***panCK*** **CTCs/mL**	***panCK+CSV*** **CTCs/mL**
001	#3	Osteosarcoma	2.5	3.0
005	#3	Osteosarcoma	0.0	0.0
007	#3	Osteosarcoma	2.5	3.0
007	#5	Osteosarcoma	0.5	1.0
008	#3	Osteosarcoma	0.5	0.5
018	#2	Osteosarcoma	0.0	1.5
037	#1	Osteosarcoma	2.5	3.5

[†]Some patients contributed more than one sample to the entire study. "Sample No." serves as an identifier to distinguish among different samples contributed by the same patient.

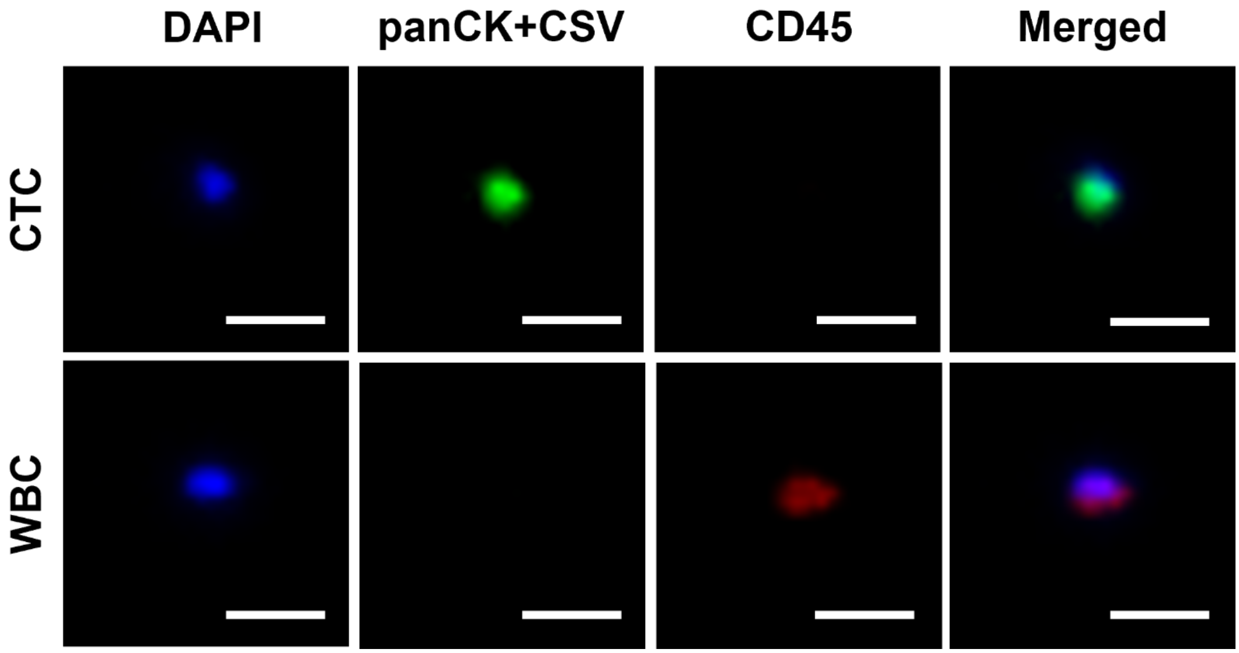

Figure S1. Representative images of a *panCK+CSV* sarcoma CTC and a WBC as detected using immunofluorescence. Here, the patient sample was subjected to the fluorescence cocktail consisting of DAPI, panCK-AF, CSV-FITC, and CD45-PE. The *panCK+CSV* CTCs were defined by the phenotype $DAPI^{+}(panCK+CSV)^{+}CD45^{-}$. The WBCs were defined as $DAPI^{+}(panCK+CSV)^{-}CD45^{+}$. Scale bars are 20 μm.

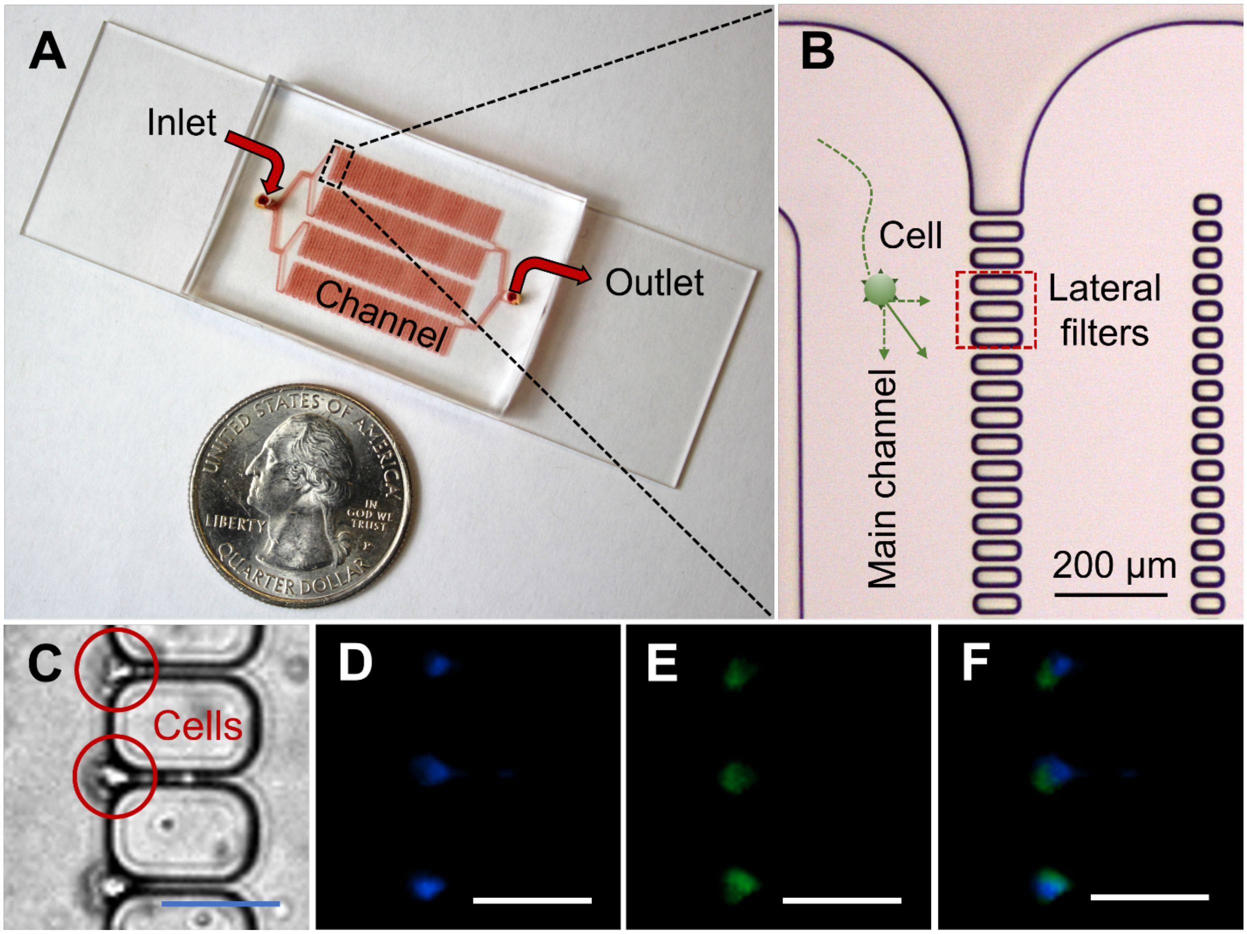

Figure S2. The LFAM chip with (A) one inlet, one outlet, and four serpentine main channels that feature (B) arrays of lateral filters. Cells traveling inside the microchannels possess velocity components both along the main channels and into the filter arrays. When functionalized with capture agents such as antibodies against GD2 and CSV, the LFAM device can capture CTCs based on both filtration and immunoaffinity. (C) Hu09 cells captured in the LFAM device visualized under brightfield microscope, (D-E) fluorescence microscope images after being stained with DAPI (blue), a mixture of panCK-Alexa Fluor 488 and CSV-FITC (both in green), and (F) the overlay of images of D and E. Scale bars for (C-F) are 50 μm.

Table S5. Comparison between applications of the LFAM microfluidic device.

Reference	Cancer type	Capture antibodies	Tumor marker for detection	Number of patients tested
[1]	Colorectal	EpCAM	CK	12
[2]	Pancreatic	EpCAM	CK	7
[3]	OS	GD2+CSV	CK	2
This work	OS, EWS, RMS, Chordoma, DSRCT, RCS, SS	GD2+CSV	CK, panCK, panCK+CSV	21

Table S6. Sarcoma patients and number of samples collected for the entire study.

Patient ID	Sarcoma Subtype	Number of Samples
001	Osteosarcoma	3
002	Rhabdomyosarcoma	2
003	Chordoma	1
004	Rhabdomyosarcoma	2
005	Osteosarcoma	3
007	Osteosarcoma	5
008	Osteosarcoma	3
013	Round Cell Sarcoma	1
014	Ewing Sarcoma	1
015	Ewing Sarcoma	1
016	Ewing Sarcoma	2
017	Ewing Sarcoma	1
018	Osteosarcoma	2
019	Ewing Sarcoma	2
022	Synovial Sarcoma	2
025	Desmoplastic Small Round Cell Tumor	3
026	Synovial Sarcoma	1
028	Synovial Sarcoma	1
031	Synovial Sarcoma	1
037	Osteosarcoma	1
041	Osteosarcoma	1

References

1 Chen, K., Dopico, P., Varillas, J., Zhang, J., George, T. J. & Fan, Z. H. Integration of Lateral Filter Arrays with Immunoaffinity for Circulating-Tumor-Cell Isolation. *Angewandte Chemie (International ed. in English)* **58**, 7606-7610, DOI: https://doi.org/10.1002/anie.201901412. Epub 2019 May 9. (2019).

2 Dopico, P. J., Le, M.-C. N., Burgess, B., Yang, Z., Zhao, Y., Wang, Y., George, T. J. & Fan, Z. H. Longitudinal Study of Circulating Biomarkers in Patients with Resectable Pancreatic Ductal Adenocarcinoma. *Biosensors* **12**, 206 (2022).

3 Fasanya, H. O., Dopico, P. J., Yeager, Z., Fan, Z. H. & Siemann, D. W. Using a combination of gangliosides and cell surface vimentin as surface biomarkers for isolating osteosarcoma cells in microfluidic devices. *Journal of Bone Oncology* **28**, 100357, DOI: https://doi.org/10.1016/j.jbo.2021.100357 (2021).
